# Evaluating Management Scenarios for the European Hamster (*Cricetus cricetus*) Using Quantitative Models

**DOI:** 10.1002/ece3.72353

**Published:** 2025-10-21

**Authors:** Imke Tomsin, Alexander Bradley Duthie, Nils Bunnefeld, Herwig Leirs, Jim Casaer, Natalie Beenaerts

**Affiliations:** ^1^ Centre for Environmental Sciences Hasselt University Hasselt Belgium; ^2^ Biological and Environmental Sciences University of Stirling Stirling UK; ^3^ Evolutionary Ecology Group University of Antwerp Antwerpen Belgium; ^4^ Research Institute for Nature and Forest (INBO) Brussel Belgium

**Keywords:** agriculture, conservation measures, European hamster, natural resource management, quantitative modelling

## Abstract

The European hamster ( *Cricetus cricetus* ) is critically endangered across its range, with modern intensive agriculture and habitat fragmentation mainly driving population declines. Conservation efforts have been largely ineffective in reversing these trends, emphasising the need for innovative approaches, such as quantitative modelling, to evaluate and guide management actions. We used the Generalised Management Strategy Evaluation (GMSE) framework to develop an individual‐based model for the European hamster. We simulated population dynamics for a population in the western part of the species' range under various hypothetical management scenarios. Twelve scenarios were tested to evaluate the impact of different life history parameters on population dynamics over 5 years. Simulations based on current conditions, including low reproduction and survival rates under intensive agriculture, predicted a steady population decline. Scenarios incorporating increased reproduction and survival within hamster‐friendly agricultural fields demonstrated varying degrees of population stabilisation and growth, with only the most optimistic projection achieving the target population size. Our simulations suggest that, under current conditions and without substantial improvement in population parameter values, potentially achievable through targeted management interventions, the European hamster is unlikely to recover in the western part of its range. Increasing the average number of litters per female per year alone is insufficient; population growth was only observed in scenarios combining improvements in multiple reproductive parameters and survival rates, which may be difficult to achieve in practice. While our model is not intended to produce exact predictions or prescriptive guidance, it offers a valuable tool for exploring hypothetical scenarios and investigating the consequences of model assumptions. As such, it can inform the design of more adaptive and ambitious conservation strategies, in line with the IPBES Scenarios and Models Assessment, which highlights the role of modelling for policy development and integrating biodiversity conservation with ecosystem services.

## Graphical abstract

We used an individual‐based model to evaluate hypothetical management scenarios for the critically endangered European hamster in Western Europe. Simulations indicate continued population decline in the absence of hamster‐friendly management, with recovery possible only through ambitious improvements in reproduction in hamster‐friendly agricultural landscapes.

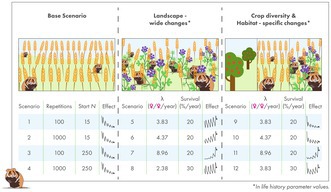

## 1 Introduction

The rapid, global decline in biodiversity, driven primarily by anthropogenic activities, is one of the most pressing issues of our time (Díaz et al. 2019). Addressing this crisis requires effective conservation and resource management strategies to prevent species extinctions and ecosystem degradation. Conservation biology and resource management are inherently complex and characterised by uncertainty and a lack of straightforward solutions (Game et al. 2014). Quantitative modelling can play an important role in addressing this uncertainty, as it can be used to simulate alternative scenarios and assess the effects of different management strategies on simulation outcomes (Milner‐Gulland and Shea 2017; Mouquet et al. 2015). Despite the recognised value of quantitative modelling and its integration into several decision‐making frameworks, such as adaptive management, structured decision‐making (SDM) and management strategy evaluation (MSE), it remains underused in biodiversity conservation and natural resource management planning (Bunnefeld et al. 2017).

The European hamster ( *Cricetus cricetus* Leske, 1779) provides a compelling case for the need to integrate quantitative models into conservation planning. Once common and regarded as an agricultural pest, the species is now critically endangered with decreasing populations throughout its range (Banaszek et al. 2019; Surov et al. 2016). Intensive agriculture is generally considered the greatest threat to the species, followed by habitat fragmentation (Bald et al. 2021; La Haye et al. 2012, 2014; Neumann et al. 2004; Rusin et al. 2013; Villemey et al. 2013). Modern machinery enables rapid harvesting across large areas in a short period, removing protective cover and food sources, thereby exposing hamsters and their burrows to predators. Additionally, harvesting causes hamsters to abandon their burrows in search of alternative habitats, further increasing predation risk (Bald et al. 2021; Bihari and Arany 2001; Müskens et al. 2005). Moreover, harvests now occur earlier than they historically did, disrupting the hamsters' reproductive cycle and reducing their overall reproductive success (La Haye et al. 2014). The use of heavy machinery and ploughing also poses direct threats such as soil compaction, burrow destruction and occasional mortality of individuals (La Haye et al. 2020; Weinhold and Kayser 2006). These impacts are considered less significant compared to the loss of protective cover and food availability. Similarly, hamsters may die due to road accidents, with the number of road‐killed hamsters generally reflecting the population density (Surov et al. 2016; Weinhold and Kayser 2006).

Habitat fragmentation threatens the species by isolating populations and reducing genetic diversity, thereby increasing their vulnerability to local extinctions. In the western part of the species' range, genetic variation is especially low, which can be attributed to small population size and past demographic bottlenecks (La Haye et al. 2012; Neumann et al. 2004).

Beyond these threats, emerging concerns such as climate change and light pollution may exacerbate the species' decline by disrupting hibernation synchronisation with food availability and habitat suitability. These factors are potentially underestimated threats requiring further study and conservation attention (Banaszek et al. 2019; Surov et al. 2016).

The hamster's conservation presents another challenge: while the species provides important ecosystem services such as prey for predators, soil aeration and the creation of microhabitats (Celebias et al. 2019; Hędrzak et al. 2021), it can also cause significant crop damage when populations reach high densities (Nechay et al. 1977). Historically, when the species was still abundant, conflicts with farmers were more pronounced. Crop damage often led to control measures, such as widespread eradication campaigns offering bounties for killed hamsters, as farmers sought to protect their incomes (Libois and Rosoux 1982; Mercelis 2002). These problems indicate the social–ecological nature of European hamster conservation and the need for management strategies that balance the conservation of the species with mitigating economic impacts.

Conservation strategies for the European hamster have typically focused on restoration and management of their habitat, which involves incentivising farmers to adopt hamster‐friendly agricultural practices that minimise harm to the species and its habitat, while remaining economically viable (Kuiters et al. 2010; La Haye et al. 2020). Examples include leaving strips of unharvested crops and managing fields of cereals and lucerne to ensure sufficient coverage throughout the active season, offering hamsters food and protection from predators (Kuiters et al. 2010). Another form of conservation that has been tested is predator exclusion. For instance, electric fencing has been shown to significantly increase survival (La Haye et al. 2020; Villemey et al. 2013). However, to date, no conservation programme has succeeded in establishing a minimum viable, stable or growing European hamster population. At best, these programmes have managed to stabilise small populations through the yearly introduction of captive‐bred individuals (Kletty et al. 2020; Surov et al. 2016; Van Donink and Baert 2023). This lack of success highlights the need for tools that can support conservation planning by allowing flexible exploration of alternative management strategies.

To contribute to this effort, we apply the Generalised Management Strategy Evaluation (GMSE) framework developed by Duthie et al. (2018). GMSE is based on Management Strategy Evaluation (MSE), originally developed in fisheries science to explore the outcomes of alternative management strategies under uncertainty, and later expanded to terrestrial conservation applications (Bunnefeld et al. 2011). GMSE extends the capabilities of MSE by integrating elements of game theory and using genetic algorithms to model decision‐making processes of managers and stakeholders.

In this study, we only use the natural resources submodel of GMSE as a foundation to build a spatially explicit individual‐based model (IBM) focused on the European hamster. This submodel simulates basic ecological processes such as reproduction, survival and movement across a landscape. We did not implement GMSE's observation, manager and user submodels, as our goal was not to simulate decision‐making processes, but to explore the effects of hypothetical management scenarios on the European hamster's population dynamics. We implemented the model within GMSE to facilitate future extensions. The framework's modular design allows for the integration of additional components (e.g., observation processes, manager policy‐making, stakeholder responses), which may become feasible as further ecological or social data become available.

We explore the potential outcomes of different hypothetical management scenarios for a European hamster population in Flanders (Belgium), where the most recent species protection programme (2015–2020) failed to achieve population growth, despite an investment of €800,000 (Agentschap voor Natuur en Bos 2015). Through simulations, we test whether population recovery could occur under assumed increases in reproductive output and survival, potential outcomes of hamster‐friendly management strategies, whose real‐world effectiveness remains uncertain. This study also marks the first application of GMSE's natural resources submodel to simulate a small, endangered population, demonstrating its flexibility for adaptation to new cases.

## 2 Methods

The European hamster model was designed using R statistical software (R Core Team 2023), and the R package GMSE (Duthie et al. 2022). The ‘gmse_apply’ function was used to enhance the individual‐based natural resources submodel and landscape layer of the GMSE model. This allowed us to tailor them specifically to the case of the European hamster and included introducing differences in life history parameters across time steps and modifying the landscape layer to simulate various crop management scenarios. Below, we briefly summarise the model and provide an overview of the differences between the simulated scenarios. A full model description following the ODD (Overview, Design concepts, Details) protocol for describing individual‐ and agent‐based models (Grimm et al. 2006), as updated by Grimm et al. (2020), is available in Appendix S1.

The purpose of this model is to explore how changes in population parameter values, assumed to be caused by hypothetical management actions, could influence the population dynamics of a European hamster population. Rather than predicting exact outcomes, the model serves as a tool to investigate the potential implications of various management strategies under simplified and controlled assumptions. Its ultimate purpose is to support conservation planning by identifying the types and magnitudes of changes that may be associated with population stability or growth. We evaluate our model by its ability to reproduce population dynamics similar to those observed in wild European hamster populations in the western part of the species' range (including Belgium, the Netherlands, Alsace, and North Rhine‐Westphalia). Population parameter values used in the model were selected from studies conducted in this part of the range.

The model simulates the population dynamics of the European hamster over a set number of time steps, where each time step represents 1 month. It follows a 12‐month cycle, beginning in January. By using monthly time steps, we aim to balance biological realism with computational efficiency, capturing the seasonal patterns relevant to the species while keeping the model tractable and interpretable. Moreover, more fine‐grained input data (e.g., weekly or daily) is often unavailable or highly uncertain.

Key model parameters were informed by available empirical data where possible and supplemented with hypothetical values to explore a range of plausible ecological responses to management scenarios. The model includes stochasticity in survival, reproduction and movement. A brief overview of European hamster life history is provided here, with full details and justifications for all parameter values in Appendix S1.

European hamsters spend part of the year overwintering, typically from October to March (Siutz et al. 2016; Weinhold and Kayser 2006; Wollnik and Schmidt 1995). The active season starts in April; then it takes about a month before females start reproducing (Franceschini‐Zink and Millesi 2008; Siutz et al. 2016). Reproductive season length varies across the species' range, beginning around May in Western Europe and about a month earlier in the east, likely due to more favourable climatic conditions (Nechay 2000; Surov et al. 2016; Weinhold and Kayser 2006). Under natural conditions, females can produce up to three litters per season. In Western Europe, they usually have one to two, while an extra litter may occur in the east due to the longer breeding season (Franceschini‐Zink and Millesi 2008; Weinhold and Kayser 2006).

Average litter size has declined from 6 to 12 offspring in the past to 3.43 today, indicating a significant reduction in reproductive output over recent decades (Nechay et al. 1977; Surov et al. 2016). This decline may be linked to reduced food availability, as higher reproductive success has been reported in females consuming more protein (Gérard et al. 2024) and in individuals living in mesocosms with greater crop diversity (Tissier et al. 2018).

In September, hamsters begin hoarding food before overwintering underground as temperatures drop (Siutz et al. 2016; Weinhold and Kayser 2006; Wollnik and Schmidt 1995). They typically live 1–2 years in the wild, and predation is the primary cause of mortality, with foxes, birds of prey and small mustelids being responsible for 73% of all deaths (La Haye et al. 2020; Nechay 2000; Nechay et al. 1977; Weinhold and Kayser 2006).

Individuals are simulated on a spatially explicit landscape, which is divided into grid cells representing areas of 100 m^2^ corresponding to hamster territories or burrow locations. Movement is modelled as the relocation of a hamster's territory and occurs after hibernation and reproduction. In addition to individual survival and reproduction, the model accounts for intraspecific competition, where only one hamster survives when two or more co‐occur on the same grid cell, mimicking territorial behaviour observed in the species (Nechay 2000). This introduces density dependence due to the limited number of grid cells in the landscape. This is the only form of density dependence included in the model, which we consider appropriate given our focus on small, minimum viable populations.

Model simulations ran for 60 time steps, corresponding to a total period of 5 years, reflecting the duration of the Flemish species protection programme (Agentschap voor Natuur en Bos 2015). Each simulation was repeated 100 times, except for Scenarios 2 and 4 (see details below). The population size at the end of each time step was recorded for each replication. Data were visualised using the ggplot2 R package (Wickham 2016).

Twelve different simulation scenarios were designed (see Appendix S1, 8: Simulation scenarios for a more detailed explanation). This allowed us to test the new model's functionality and assess how, under modelled assumptions, the types of measures proposed in the Flemish species protection plan might influence population dynamics. Table 1 shows an overview of differences between simulated scenarios. Scenarios 1–4 were designed to test the impact of different starting population sizes (15 and 250) and to compare the average population size over time using 100 and 1000 replications. Life history parameter values were kept the same across these scenarios, representing a base scenario that aims to reflect typical conditions for European hamster populations in Western Europe without additional (management) interventions or extreme conditions. This base scenario serves as a standard for comparing the effects of parameter adjustments and alternative scenarios. Scenarios 5–8 introduced variations in reproductive output and survival probability across the entire landscape to assess their effects on population dynamics. These scenarios tested different levels of reproductive success, from modest increases in litter size (Scenarios 5 and 6) to higher reproduction rates based on historical data (Scenario 7), as well as a 10% increase in survival probability (Scenario 8). Scenarios 9–12 introduced crop diversity in the landscape, and variations in reproductive output and survival rates of European hamsters specific to each crop type. Three hypothetical crop types, representing different levels of habitat suitability, were defined (see Appendix S1, Table S4 for rationale and references). Crop type 1 represented unsuitable habitat where hamster survival was not possible, crop type 2 was the same as the base scenario (Scenarios 1–4), and crop type 3 represented hamster‐friendly agriculture, characterised by increased average reproductive output or survival rates, similar to Scenarios 5–8. At the start of each replication, the spatially explicit landscape was divided into 10 fields, each belonging to a different stakeholder. This setup provides a simplified representation of agricultural landscapes, which in reality are often more heterogeneous. Crop types were distributed randomly over these fields, but a predetermined number of fields were assigned each crop type, with four fields receiving crop type 1, another four receiving crop type 2, and two receiving crop type 3 (See Figure S1 in Appendix S1 for an example). According to the Flemish species protection plan, some form of hamster‐friendly management (represented here by crop type 3) would be required on at least 20%–25% of fields to achieve a favourable conservation status for the European hamster, although this threshold is not strongly justified in the document and does not appear to be based on empirical data (Agentschap voor Natuur en Bos 2015). We adopted this proportion in our model to reflect the plan's recommendation and to explore its potential effectiveness under simulated conditions.

**TABLE 1:** Overview of parameter differences between and results of all 12 simulation scenarios, modelled using GMSE (Duthie et al. 2018).

Scenario	Reps	Initial pop. size	Lambda (♀/♀/year)	Survival rate (%/year)	Crop diversity	Mean pop. size (*t* = 60)	SD (±)
1	100	15	2.38	20	No	1.55	2.79
2	1000	15	2.38	20	No	1.92	3.70
3	100	250	2.38	20	No	38.54	12.10
4	1000	250	2.38	20	No	39.81	14.83
5	100	250	3.83	20	No	260.93	67.58
6	100	250	4.37	20	No	483.79	103.77
7	100	250	8.96	20	No	10,078.21	1648.06
8	100	250	2.38	30	No	234.19	53.12
9	100	250	CT2: 2.38 CT3: 3.83	CT1: 0 CT2‐3: 20	Yes	37.52	21.40
10	100	250	CT2: 2.38 CT3: 4.37	CT1: 0 CT2‐3: 20	Yes	56.40	26.18
11	100	250	CT2: 2.38 CT3: 8.96	CT1: 0 CT2‐3: 20	Yes	852.43	398.75
12	100	250	CT2: 2.38 CT3: 3.83	CT1: 0 CT2: 20 CT3: 30	Yes	150.09	76.56

*Note:* The number of replications run (reps) and initial European hamster population size for each scenario are shown. Each simulation includes monthly time steps, with lambda values divided over June and July in the base scenarios (1–4). For scenarios involving an increased lambda (5–7 and crop type 3 in 9–12), lambda is divided across June, July, and August. See Tables S2 and S3 for details on monthly survival rates used in simulations. The ‘Crop diversity’ column indicates whether the landscape is uniform (No) or includes different crop types with varying population parameters based on individuals' locations (Yes). The mean population size reached at the end of the replications (after 60 time steps, i.e., months) and standard deviation (SD) are shown. For more information and references about parameter choices, see Appendix S1.

## 3 Results

**FIGURE 1:**
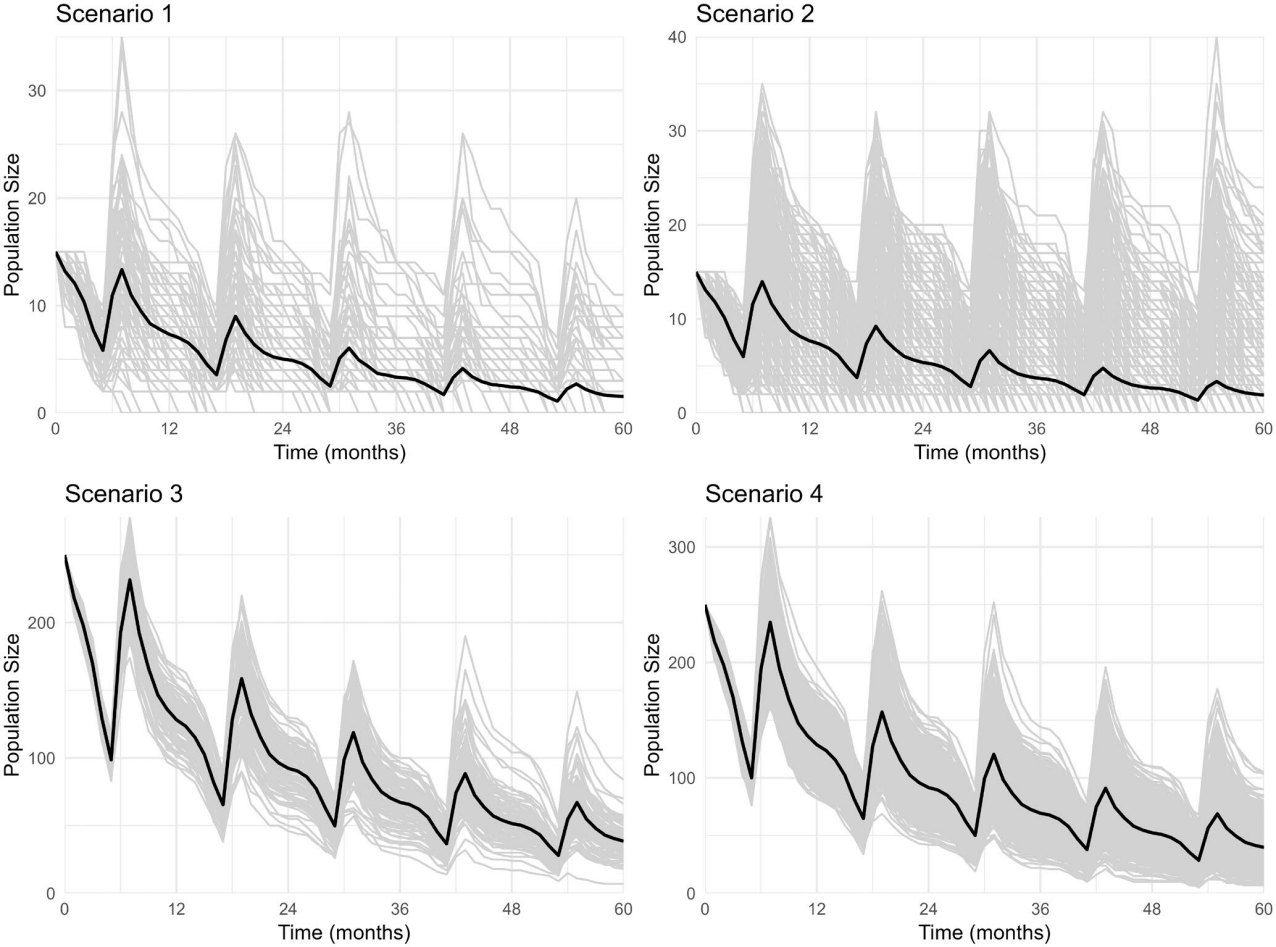
European hamster population size over time for the four base scenarios (Scenarios 1–4), modelled using GMSE (Duthie et al. 2018). Individual simulation runs are shown in grey, and the mean population size across replicate simulations is shown in black. Time steps represent 1 month. Scenario 1: 100 replications with an initial population size of 15, Scenario 2: 1000 replications with an initial population size of 15, Scenario 3: 100 replications with an initial population size of 250, Scenario 4: 1000 replications with an initial population size of 250.

We observed fluctuations in population size in all simulations, characterised by a yearly peak following the reproductive season, followed by a gradual decline until the start of the next reproductive season. The first four scenarios show a marked decline in mean population size over time, with strong seasonal fluctuations (Figure 1). At the end of the simulations (time step 60), the mean (±SD) population sizes for all replications were as follows: Scenario 1–1.55 (±2.79), Scenario 2–1.92 (±3.70), Scenario 3–38.54 (±12.10), and Scenario 4–39.81 (±14.83) individuals. An overview of the parameter differences and results for all 12 simulation scenarios is provided in Table 1.

Extinction was defined as the population size reaching zero at any time during the simulation. In Scenarios 1 and 2, with an initial population size of 15, extinction probabilities were 72.0% and 69.8%, respectively. Scenarios 3 and 4, with an initial population size of 250, showed no extinction events, but population sizes consistently declined over time, approaching levels indicative of long‐term unsustainability.

**FIGURE 2:**
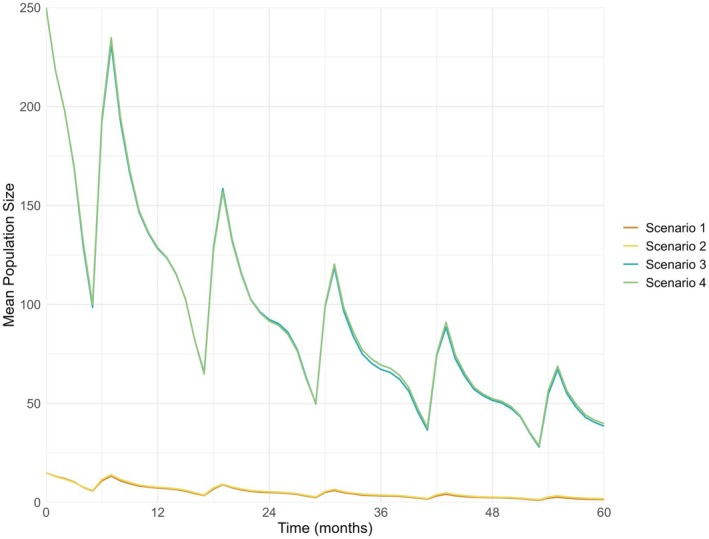
Mean European hamster population size over time compared for Scenarios 1–4, modelled using GMSE (Duthie et al. 2018). Time steps represent 1 month. Scenario 1: 100 replications with an initial population size of 15, Scenario 2: 1000 replications with an initial population size of 15, Scenario 3: 100 replications with an initial population size of 250, Scenario 4: 1000 replications with an initial population size of 250.

Comparison of the mean population size over time of Scenarios 1–4 shows that increasing the number of replications from 100 to 1000 has minimal impact on the overall trend for both initial population sizes, indicating that the model's predictions are relatively robust to the number of replications (Figure 2).

**FIGURE 3:**
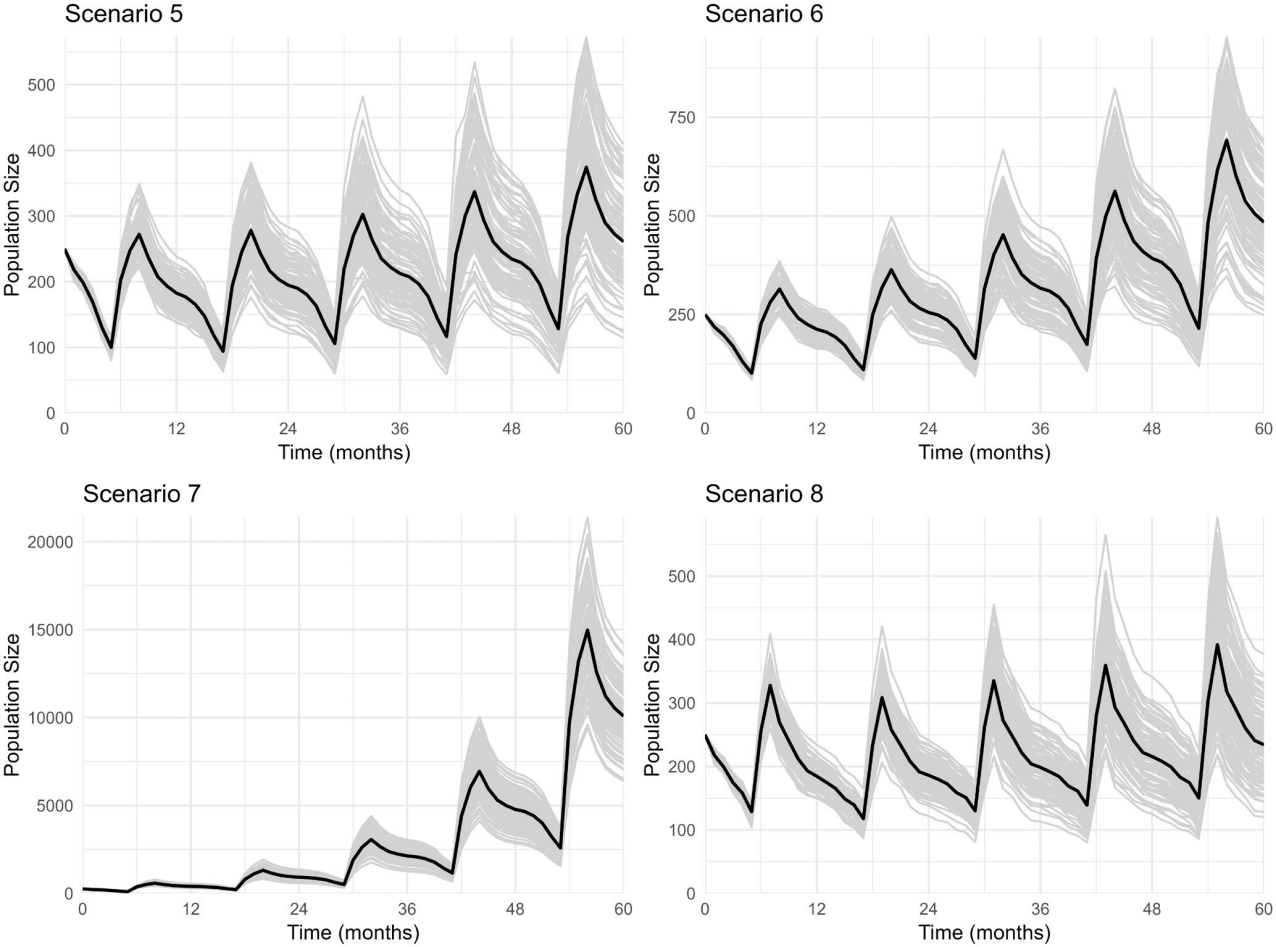
European hamster population size over time for Scenarios 5–8, modelled using GMSE (Duthie et al. 2018). Individual simulation runs are shown in grey, and the mean population size across replicates is shown in black. Time steps represent 1 month. These scenarios model differences in reproduction and survival probability to examine their effects on European hamster population growth. Scenario 5: Increase of average number of litters with 1, Scenario 6: Increase of average number of litters with 1.37, Scenario 7: Increase of average number of litters with 0.93 and average number of pups per litter with 4.81, Scenario 8: Increase of mean annual survival with 10%.

In Scenario 5, the mean population size of all replications at the end of the simulation period was 260.93 individuals (±67.58), which is close to the initial population size of 250. In Scenario 6, the mean population size after 5 years increased to 483.79 individuals (±103.77), nearly doubling the initial population. In Scenario 7, a mean final population of 10,078.21 individuals (±1648.06) was reached, a more than 40‐fold increase over the initial population size. Scenario 8 resulted in a mean population size of 234.19 individuals (±53.12), which was slightly below the initial size. These results are illustrated in Figure 3.

**FIGURE 4:**
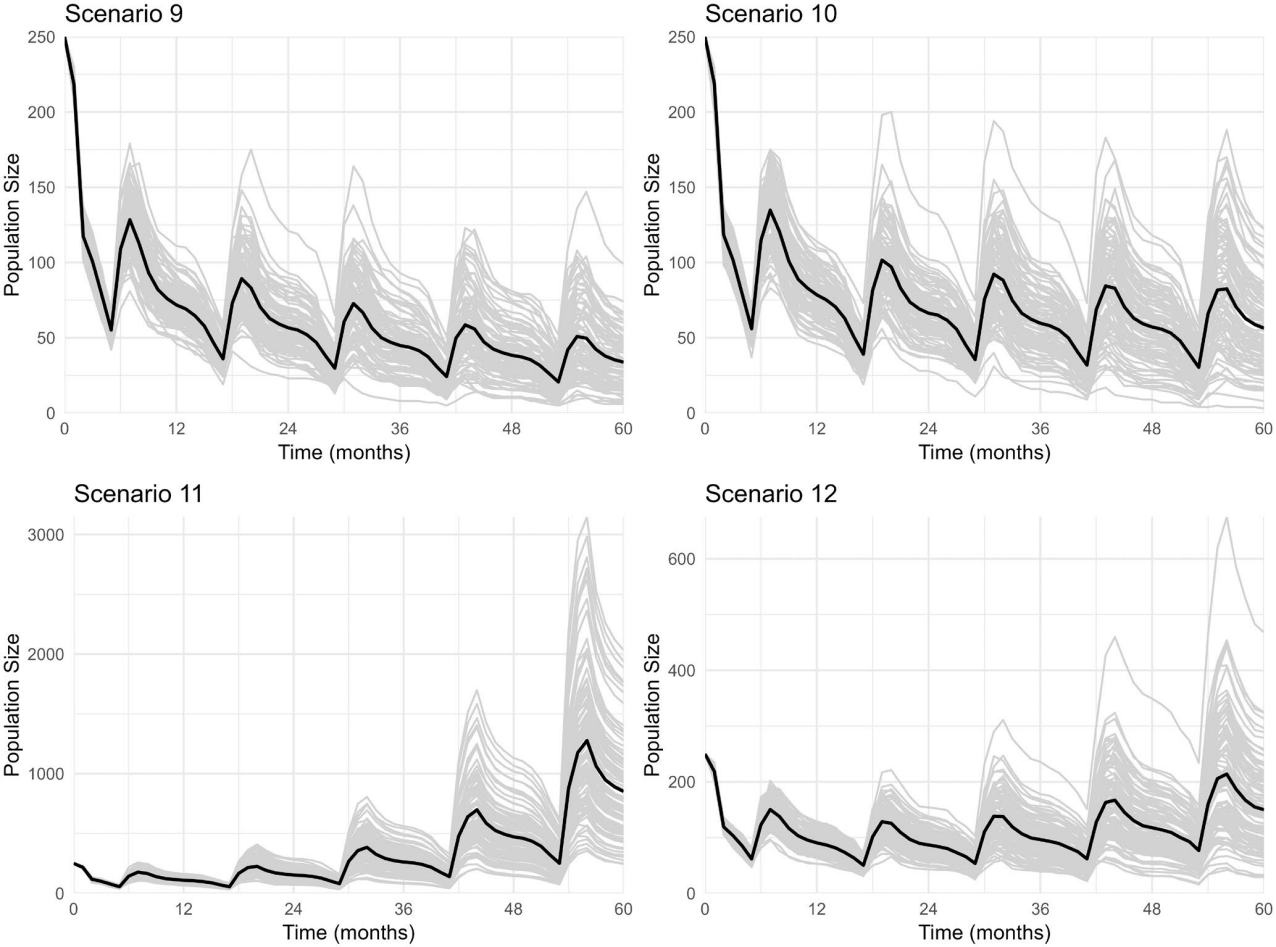
European hamster population size over time for Scenarios 9–12, modelled using GMSE (Duthie et al. 2018). Individual simulation runs are shown in grey, and the mean population size across replicates is shown in black. Time steps represent 1 month. These scenarios model differences in reproduction and survival probability to examine their effects on European hamster population growth in a landscape with different crop management strategies, where crop type 3 represents hamster‐friendly agriculture. The landscape was divided in 10 fields of the same size. Crop types were distributed randomly over these fields, but a predetermined number of fields were assigned each crop type, with four fields receiving crop type 1, another four receiving crop type 2, and two receiving crop type 3. Scenario 9: Increase in the average number of litters with 1 in crop type 3, Scenario 10: Increase in the average number of litters with 1.37 in crop type 3, Scenario 11: Increase in the average number of litters with 0.93 and average number of pups per litter with 4.81 in crop type 3, Scenario 12: Increase in the average number of litters with 1 and the mean annual survival with 10% in crop type 3.

In Scenario 9, the mean population size of all replications at the end of the simulation period was 37.52 individuals (±21.40), substantially lower than the initial population size of 250 individuals. In Scenario 10, this was 56.40 individuals (±26.18), also lower than the initial value, with the rate of decline decreasing towards the end of the simulations. In Scenario 11, the population reached a mean of 852.43 individuals (±398.75). Notably, just before the start of reproduction in Year 5 of Scenario 11, the population size averaged 251.38 individuals (±114.67), aligning closely with the target population size of 250 individuals. In Scenario 12, the final mean population size was 150.09 individuals (SD = ±76.56), following an initial decline and a gradual increase starting around Year 3. These results are illustrated in Figure 4. In these scenarios, the decrease in population size after the first time step was relatively large compared to subsequent fluctuations observed during the simulation period.

## 4 Discussion

The objective of our research was to develop a quantitative model to explore the viability of a European hamster population in the western part of the species' range under different hypothetical management scenarios. Using the GMSE framework with a modified landscape layer, we simulated how plausible changes in population parameter values, associated with conservation interventions, could affect population dynamics. Specifically, we examined whether the goals that were outlined in the Flemish species protection programme (2015–2020) could have helped stabilise or reverse population decline.

Our results suggest that, in the absence of significant management interventions, population decline remains likely. Population growth was only observed in scenarios that assumed substantial increases in both reproductive output and survival within hamster‐friendly managed areas. These results demonstrate the value of model‐based scenario testing to help guide more ambitious and targeted conservation strategies for this endangered species.

### 4.1 European Hamster Viability Under Different Scenarios

Our results predict a consistent population decline under the base scenario (Scenarios 1–4), which represents a business‐as‐usual approach without additional management. This decline leads to extinction, albeit over a longer period than 5 years depending on initial population size.

Scenarios with increased reproduction compared with the base scenario (Scenarios 5–7) led to population growth, but achieving such parameters without widespread management seems unlikely. An increase in survival alone (Scenario 8) reduced the rate of decline but did not reverse it.

To assess the feasibility of the Flemish species protection programme's goal of 500 individuals after 5 years, we tested four different scenarios (Scenarios 9–12) where 20% of the landscape was under hamster‐friendly management, reflecting a longer‐term goal outlined in the programme. This was simulated by improving population parameters for individuals located in hamster‐friendly fields. Increasing the average yearly number of litters per female in hamster‐friendly fields, which in reality might be achieved by delaying harvests until after the reproductive season (La Haye et al. 2014), was insufficient to achieve population growth within the simulation period. Only when both average litter size (8.24 pups per litter) and the average number of litters (2.56 per female per year) were increased (Scenario 11), reflecting mean annual reproductive rates of female European hamsters before 1985 (Surov et al. 2016), did the population exceed its initial size after 5 years. An average population size of 250 individuals was reached in this scenario before the start of the breeding season in Year 5. Of all scenarios that involved crop diversity in the landscape, this is the only one in which the species protection programme target was met within 5 years. Scenario 12 also showed a positive trend after an initial decline, potentially due to more hamsters occupying hamster‐friendly managed habitat over time.

The declines in population size observed in Scenarios 9–12 after the first time step likely resulted from the initial random placement of individuals across the landscape, including in unsuitable habitat (crop type 1), where individuals are removed from the population at the end of the time step. While initialising all individuals in suitable habitat (crop types 2 and 3) could be explored in future versions, the presence of unsuitable habitat reflects current landscape realities, as some designated conservation land is unsuitable for the European hamster (e.g., livestock farms, greenhouses, orchards).

### 4.2 Conservation Implications

Our simulations suggest that managing 20% of the designated conservation area in a hamster‐friendly way (Scenarios 9–12), as proposed in the Flemish species protection programme, could support population growth and help approach a minimum viable population. However, these positive outcomes were only observed under optimistic assumptions. Specifically, only when reproductive parameters were increased to pre‐1985 levels in hamster‐friendly areas (Scenario 11) did the population exceed its initial size at the end of the simulation period.

It is important to note that these model outcomes are based on simplified conditions and should be interpreted as hypothetical projections. For example, the model assumes an initial population size of 250 individuals and the immediate presence of hamster‐friendly management on 20% of the landscape. These are conditions that do not currently reflect reality. In practice, achieving these conditions would likely require the release of a substantial number of captive‐bred individuals, considering only a handful of European hamsters currently remain in the study area (J. Ramaekers, personal communication).

Additionally, our model assumes chosen parameter values reflect those of wild European hamsters or reintroduced individuals that have successfully settled. However, the population in Belgium heavily relies on the release of captive‐bred individuals, and their population parameter values may not align with those used in the model. Monitoring data indicate higher mortality shortly after release, although survival improves after an initial adaptation period (La Haye et al. 2020).

A key question remains as to how real‐world hamster‐friendly management actions will affect survival and reproduction. Our simulations indicate that ambitious and well‐targeted policies may be necessary to achieve population growth within the short term of a species conservation programme. Importantly, an increase in the number of litters alone appears insufficient. Improvements in survival and/or litter size may also be required. We recommend that future implementations of hamster‐friendly agricultural practices be accompanied by close monitoring of population parameters to evaluate whether these interventions yield the intended effects. Integrating these monitoring data with local farming knowledge could form the basis for an adaptive management framework, enabling policymakers, conservationists and farmers to learn about the social–ecological system and better balance conservation with food production (Cusack et al. 2022; Månsson et al. 2023).

### 4.3 Model Trade‐Offs and Limitations

While our results suggest that management actions enhancing survival and reproduction could facilitate short‐term population growth, these findings must be considered within the limitations of the model. One key limitation is the lack of genetic factors, which are especially important for small populations. Genetic drift and inbreeding depression can substantially increase extinction risk (Charlesworth and Charlesworth 1987; Lacy 1997), but these processes are not currently represented in GMSE. As a result, population projections at low densities may be overly optimistic. To partially address this, we used a starting population of 250 females, aligning with the Flemish species protection programme goal and approximating the 50/500 rule for maintaining genetic viability given the 1:1 sex ratio at birth. While this threshold reduces concern about genetic factors at the start of the simulation, our results frequently showed population declines to much lower levels, where in reality inbreeding and genetic drift could significantly amplify declines.

Another model limitation is the lack of environmental stochasticity, such as climate fluctuations, natural disasters and changes in resource availability, which can significantly affect population dynamics (Lande 1993). Its absence may lead to an underestimation of the system's variability and the population's extinction risk. Future extensions of GMSE could benefit from incorporating stochastic processes to better reflect real‐world conditions.

Another trade‐off of the GMSE model is its use of a single parameter, ‘lambda’, to represent reproduction. While this simplifies parameterisation, it reduces the complexity of natural reproductive strategies, which can be influenced by factors such as age structure, sex ratios and individual health. To address this, our lambda estimates incorporate average litter size, proportion of females reproducing and sex ratio for a more realistic approximation of reproductive output.

### 4.4 Broader Contributions to Modelling and Policy Relevance

The use of quantitative models, as demonstrated here, can contribute to the development of better‐informed protection programmes and natural resource management. While exact predictions cannot be made due to model assumptions and parameter variability, valuable insights can be derived by comparing trends observed between simulated scenarios (Milner‐Gulland and Shea 2017; Starfield 1997; Travers et al. 2019). Using GMSE, even without employing all its submodels, encourages consideration of stakeholders and managers and the potential to simulate their actions, which may lead to a broader understanding of the modelled issue within a social‐ecological context. It also allows for further model development as more information becomes available. Thus, even with inherent uncertainties, our model demonstrates that GMSE offers a practical and flexible framework for exploring management trade‐offs and supporting adaptive, evidence‐based conservation planning.

## Author Contributions

**Imke Tomsin:** conceptualization (lead), data curation (lead), investigation (lead), methodology (equal), project administration (lead), software (equal), supervision (equal), visualization (lead), writing – original draft (lead), writing – review and editing (lead). **Alexander Bradley Duthie:** conceptualization (supporting), data curation (supporting), investigation (supporting), methodology (equal), resources (lead), software (equal), validation (lead), writing – original draft (supporting), writing – review and editing (supporting). **Nils Bunnefeld:** conceptualization (supporting), methodology (equal), resources (supporting), validation (equal), writing – original draft (supporting), writing – review and editing (supporting). **Herwig Leirs:** conceptualization (supporting), methodology (supporting), resources (supporting), supervision (equal), writing – original draft (supporting). **Jim Casaer:** conceptualization (supporting), methodology (supporting), validation (supporting), writing – original draft (supporting), writing – review and editing (supporting). **Natalie Beenaerts:** conceptualization (supporting), investigation (supporting), methodology (equal), project administration (supporting), resources (supporting), supervision (equal), validation (supporting), writing – original draft (supporting), writing – review and editing (supporting).

## Conflicts of Interest

The authors declare no conflicts of interest.

## Supporting information


**Appendix S1:** Model description following the ODD protocol.

## Data Availability

The simulation code and data supporting this study are available at https://doi.org/10.5281/zenodo.17256240.
