# Supplementary material for: Evaluating Management Scenarios for the European Hamster (Cricetus cricetus) Using Quantitative Models
Source: Ecol Evol. 2025 Oct 21;15(10):e72353. doi: 10.1002/ece3.72353 (PMC12540919; doi:10.1002/ece3.72353)
Supplement: Supplementary file 1 — Appendix S1: Model description following the ODD protocol. [file ECE3-15-e72353-s001.docx]

# Supplementary Material 1: Overview, design concepts, and details (ODD)

Below, we describe the model used in the paper ‘Evaluating management actions for the European hamster (*Cricetus cricetus*) using quantitative models’. This model description follows the ODD (Overview, Design concepts, Details) protocol for describing individual- and agent-based models (Grimm et al., 2006), as updated by (Grimm et al., 2020).

## Purpose

The purpose of this model is to explore how changes in population parameter values, assumed to be caused by hypothetical management actions, could influence the population dynamics of a European hamster population in the westernmost part of the species’ range. Rather than predicting exact outcomes, the model serves as a tool to examine the potential implications of various management strategies under simplified and controlled assumptions. Its ultimate purpose is to support conservation planning by identifying the types and magnitudes of interventions/parameter changes that may be associated with population stability or growth.

We evaluate our model by its ability to reproduce population dynamics similar to those observed in wild European hamster populations in the westernmost part of the species’ range (incl. Belgium, the Netherlands, and Germany). Population parameters in the model were all selected from studies conducted in this part of the range (La Haye et al., 2020; Nechay, 2000; Nechay et al., 1977; Weinhold & Kayser, 2006).

## Entities, state variables, and scales

Each agent within the natural resources submodel represents one female European hamster. Individual hamster’s traits include its age in months and its specific location within the landscape, denoted by x and y coordinates.

A single time step is modelled as one month. The simulations cover a period of 60 months, with events taking place across a grid landscape. The simulated landscape spans 447 by 447 cells, equivalent to approximately 20 km². Each cell, measuring 10 by 10 m, can be occupied by a single hamster.

Each cell within the landscape has several properties. These include its geographic location (defined by x and y coordinates), ownership (indicating which farmer it belongs to), and the type of crop cultivated on it. In scenarios 9-12 (see Supplementary Material 1 – 8. Simulation scenarios), the landscape is divided among ten farmers. Each farmer owns a continuous rectangular portion of the landscape, cultivating one of three different crop types.

Crop types are assigned to farmers randomly, but the overall distribution remains constant: four areas are allocated to crop type 1, four to crop type 2, and two to crop type 3. Figure S1 illustrates an example of how crop types might be distributed across the landscape in one simulation replication.


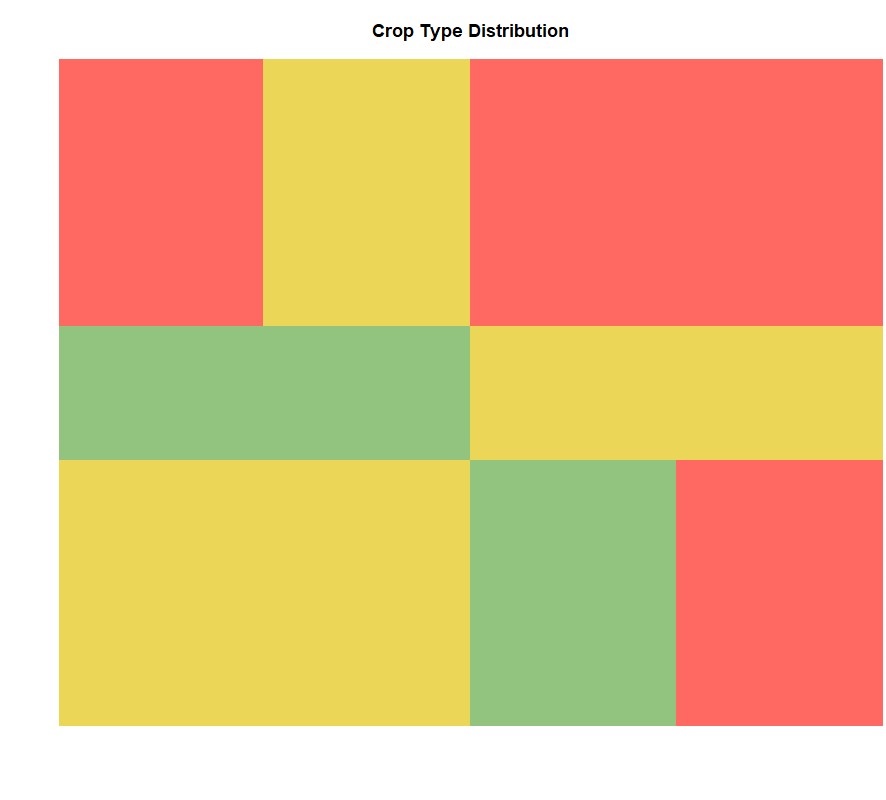

Figure S1. Example of crop type distribution across the landscape in one simulation replication, modelled using GMSE. Each coloured block represents a continuous area cultivated with a specific crop type by an individual farmer. Crop type 1 is shown in red, crop type 2 in yellow, and crop type 3 in green.

## Process overview and scheduling

The GMSE model consists of four distinct submodels that are run in sequence, and repeated across each time step:

The natural resources submodel is an agent-based model that simulates the population dynamics of the European hamster population. As population parameters change over the different time steps, movement and reproduction are only simulated in certain months. At the end of each time step, each individual’s age increases by 1. Whenever agents become 24 time steps old (i.e., 2 years), they die of old age and are removed from the population.

The observation submodel simulates the monitoring process and subsequent estimation of the natural resource population size. Resources are counted row by row across the landscape with complete accuracy. However, as manager and user actions are turned off in our model, this has no effect on our simulations.

The manager submodel simulates decision-making processes undertaken by managers (policy makers). The manager thereby attempts to set policy for stakeholders that will result in the managed species’ estimated population size being as close as possible to a target population size set by the manager. This involves determining the suitability of different management actions (e.g., culling, scaring, supplementary feeding etc.) to reach this target, alongside evaluating the cost associated with each action. In our simulations, however, managers do not attempt to set policy.

The user submodel simulates individual stakeholders, in this case farmers, that can act in ways that can affect the population size of the resource. Stakeholders must operate within the confines of policies delineated by managers. In our model, stakeholders do not attempt to influence the European hamster population size. In Scenarios 9-12, each stakeholder has ownership over a certain part of the landscape, containing a certain crop type that is kept unchanged throughout the simulation.

## Design concepts

**Basic principles**

GMSE is based on Management Strategy Evaluation (MSE), a framework that originates from fisheries management (Bunnefeld et al., 2011). GMSE extends the capabilities of MSE by integrating elements of game theory and using genetic algorithms to model decision-making processes of managers and stakeholders. GMSE thereby simulates multiple steps of a structured decision-making process (Hemming et al., 2022). Based on a certain goal, it develops alternative management actions, estimates the consequences of the different actions, evaluates the trade-offs between these consequences, and decides which action to take to reach the goal. GMSE has already been used successfully to investigate conceptual and applied questions in species management and people’s decision-making (Bach et al., 2022; Cusack et al., 2020; Duthie et al., 2018; Nilsson et al., 2021).

For this model, only the natural resources submodel and landscape of GMSE are used. Additional functions are written to adjust them for the purpose described above. The other three GMSE submodels are not used.

**Stochasticity**

Stochasticity is incorporated throughout the natural resources submodel to introduce realistic variability in reproduction, survival, and movement. The average number of offspring produced per individual is represented by the rate parameter ‘lambda,’ with the actual number of offspring for an individual in a certain time step sampled from a Poisson distribution with lambda as the rate parameter. Survival is also treated stochastically, where each individual’s survival is determined probabilistically according to monthly survival rates from La Haye et al. (2020). Movement within the landscape is modelled by letting individuals relocate up to 100 cells (i.e., approximately one kilometre) from their previous location, with direction and distance randomly sampled from a uniform distribution within this range.

Additionally, there is randomness in the initial distribution of crop types among contiguous fields in the landscape. A predetermined number of fields are assigned to each crop type, but the specific fields receiving each type are chosen randomly.

**Emergence**

In Scenarios 9-12, the model’s outcomes (i.e., the European hamster abundance at each time step) emerge from the spatial distribution of the individuals over the different crop types.

**Interaction**

When multiple (adult) hamsters attempt to occupy the same landscape cell, the model incorporates a competitive mechanism where only one competitor survives. The survivor is determined randomly, with each individual having an equal probability of being selected (i.e., a fair lottery). This mimics the observed outcomes of such interactions in nature, as European hamsters are solitary and can be very aggressive towards conspecifics. Fights between them typically result in injuries and, sometimes, cannibalism (Weinhold & Kayser, 2006). This function ensures that each cell can accommodate only one hamster at a time, establishing a carrying capacity of approximately 100 hamsters per hectare (i.e., 100 hamsters per 100 cells).

**Adaptation, objectives, learning, prediction, sensing, observation, and collectives**

Not applicable

## Initialisation

Each time step in the model represents one specific month of the year. Different parameters for reproduction, movement, and survival specific to each time step are specified at the start of a simulation, and simulations start in January. At the start of each simulation, the initial individuals of the population are distributed randomly over the landscape.

The starting population size in scenarios 1 and 2 was 15 individuals. This reflects the estimated 30 hamsters remaining in the area, with approximately 50% being females, simulating a recent situation in Belgium (Smeets, 2022). In all other scenarios, a starting population size of 250 individuals was used, reflecting a total of ca. 500 individuals in the population. It is generally argued that in the short term, a springtime population size of 500 is needed to combat inbreeding depression (Kuiters et al., 2010; Van Donink & Baert, 2023). This target was also set in the Belgian 2015-2020 species protection plan, which we evaluated using our model (Agentschap voor Natuur en Bos, 2015).

In scenarios 9 to 12, at the start of each simulation, the landscape is divided into ten continuous blocks, that each belong to a different stakeholder (i.e., there are 10 stakeholders). Crop types are distributed randomly over the blocks, but it is made sure that there are four blocks of crop type 1, four blocks of crop type 2, and two blocks of crop type 3.

## Input data

Not applicable.

## Submodels

Tables S1 and S2 provide an overview of the monthly population parameters used for the initialisation of the natural resource submodel and landscape to simulate the base scenario. Only parameters that differ from GMSE’s default settings are described below. Default values were retained where no changes were necessary. The following subsections provide a detailed explanation of the rationale behind each parameter selection, including their sources and references. All possible GMSE management actions (e.g. culling, scaring, feeding…) were disabled in our simulations. Hence, other GMSE submodels did not influence our simulation outcomes, and are not discussed further. More information on the different crop types simulated on the landscape, and an overview of values used in all simulation scenarios can be found below (See 8. Simulation scenarios).

### 7.1 Reproduction

In the natural resources submodel, the average number of offspring produced by an individual in one time step is represented by the parameter ‘lambda’. During each time step, the number of offspring produced by a resource is sampled from a Poisson distribution with lambda as the rate parameter. In our model, lambda was set to zero during time steps when hamsters do not reproduce.

In the base scenario, individuals produce offspring at the start of the months of June and July. Offspring are considered to be one time step old and weaned by the end of the time step in which they are born. The value for lambda is based on the study by Surov et al. (2016), which found that female European hamsters produce, on average, 3.43 pups per litter and 1.63 litters per year in recent decades. With a sex ratio of 1:1, only half off the offspring is female. Furthermore, only 85% of females manage to reproduce. Based on this information, lambda (λ) was calculated as follows:

λ = 3.43 offspring/litter * 1.63 litters/year * 0.5 female proportion * 0.85 females reproducing

λ ≈ 2.38 female offspring/female/year.

To simulate this over two separate months, this annual lambda value was divided by two, resulting in a lambda of 1.19, which was applied in the months June and July.

In general, it is unclear whether or not hamsters in the western part of the species’ range can already produce offspring in the year of their birth, and if so, whether this involves a high proportion of these young hamsters. In a study on the reproduction of hamsters from a West-German population, Mohr et al. (1973) found that female hamsters born in spring reach sexual maturity after about 80 days, corresponding to at least two time steps in our model. Considering a pregnancy lasts about 21 days (Mohr et al., 1973; Nechay et al., 1977), the earliest a hamster could produce offspring is when it is around 101 days old, i.e., when it is between three and four time steps old. In the base scenario, this was set at four time steps, therefore, individuals are not capable of producing offspring in the year of birth. In Nechay (2000), it is also mentioned that reproduction by offspring in the year of birth has not been observed in the western part of the range, while in the eastern part it has. In general, however, this is a parameter about which there is still uncertainty.

### 7.2 Mortality

Monthly survival rates used in the model are shown in Table S2. These survival rates are based on a large-scale study conducted in the Netherlands from 2002 to 2016, which investigated the causes of mortality and survival rates of more than 800 hamsters implanted with radio transmitters. Among these were 276 wild-born female hamsters, for which a yearly survival rate of 20% was found (La Haye et al., 2020). A similar yearly survival rate of 20% has also been documented for juveniles (Surov et al., 2016).

The survival rate of introduced captive-bred individuals is generally much lower, but reliable data on this are lacking. Therefore, the model is solely based on the survival rates of wild-born individuals or introduced individuals that have already successfully established in the wild, as survival rates equalise a few weeks after introduction, which must be considered when interpreting the results (La Haye et al., 2020).

In the western part of their range, European hamsters typically live up to a maximum of two years (Nechay, 2000; Nechay et al., 1977; Weinhold & Kayser, 2006). Therefore, the maximum age for a hamster is set at 24 time steps, meaning that an individual reaching this age will be removed from the population.

### 7.3 Landscape

To determine the size and dimensions of the GMSE model landscape (land_dim_1 and land_dim_2), we defined how the size of individual grid cells should relate to that of the overall landscape. The size of a cell was chosen to approximately match the territory of a single hamster. Several attempts have been made to estimate hamster territory size, all yielding different results. In general, the home range of a hamster seems to be inversely correlated with population density in a given area (Weinhold & Kayser, 2006). Historically, densities of 10 to 80 European hamsters per hectare were observed in typical years, but could be much higher during outbreak years. Based on this, a territory size of 10 m x 10 m was chosen as the basis for modelling. This 100 m² area corresponds to one landscape cell within the model, allowing for a density of maximum 100 individuals per hectare. The area for which the Flemish government developed the species protection programme of 2015-2020 is roughly 2,000 hectares or 20 km² (Agentschap voor Natuur en Bos, 2015). Considering a cell size of approximately 100 m², we therefore opted for a square landscape of 447 by 447 cells, resulting in a total simulated area of 199,809 grid cells.

### Movement

Individual movement is defined as the number of cells from their currently occupied cell (Chebyshev, i.e., “kings” distance) that an individual moves within a time step. This movement is modelled as the change in the location of a hamster’s burrow. In the model, burrow relocation happens after hibernation and after reproduction, specifically in April, June, and July. Hamsters regularly change burrows, and certain events in their life cycle trigger this behaviour. Before overwintering, hamsters construct a winter burrow. Upon emergence from hibernation in early spring, they abandon the winter burrow and establish a new one elsewhere. Another burrow change occurs after females have finished weaning their young, typically when the offspring are approximately three to four weeks old. Both the mother and the young will leave the old burrow, and each establish a new one. This relocation occurs after each litter (Górecki, 1977; Kayser, 2001).

Movement is simulated by allowing individuals to relocate up to a maximum of 100 cells away from their previous burrow, corresponding to a distance of one kilometre. In reality, most hamsters do not move that far, with a German study finding an average migration distance of 191 metres for females (Millesi et al., 2008). In Kuiters et al. (2010), an average distance of 132 metres was reported for females, though movement up to 1.5 kilometres away has also been observed. Similarly, in Van Wijk et al. (2011), females were found to have travelled distances ranging from 15 to 400 metres, with possible outliers exceeding one kilometre. During the monitoring period in Belgium, the furthest distance travelled by a female was 847 metres, indicating that long-distance travel is possible (Descamps & Ramaekers, 2020). This possibility was therefore included in the model.

### 7.5 Intraspecific competition

When multiple hamsters attempt to occupy the same landscape cell, the model incorporates a competitive mechanism where only one of the competitors survives, determined randomly. This mimics the observed outcomes of such interactions in nature, as European hamsters are solitary and can be very aggressive towards conspecifics. Fights between them typically result in injuries and, sometimes, cannibalism (Weinhold & Kayser, 2006).

This functionality is not inherent to the GMSE model but was added through a custom function. It is executed in the time step following one with movement, as movement occurs only at the end of each time step. This ensures that each cell can accommodate only one hamster at a time, establishing a carrying capacity of approximately 100 hamsters per hectare (i.e., 100 hamsters per 100 cells). This is the only form of density-dependence included in the model, which we consider appropriate given the study’s focus on small, minimum viable population sizes.

Agricultural land use in this area was investigated using the 2023 agricultural land use data available on the website of the Flemish government (Agentschap Landbouw en Zeevisserij, 2023). Based on GIS calculations, we found that there are approximately 1,122 individual fields in the area, with an average size of roughly two hectares.

Table S1. Overview of parameters and values used for the natural resources submodel and landscape of the base scenario (1-4) of the GMSE model for the European hamster.

| GMSE parameter | Parameter explanation | Parameter value |
| --- | --- | --- |
| lambda | Poisson rate parameter for the number of offspring produced by a resource during a time step | 1.19 (in June and July) (based on Surov et al. (2016)) |
| age_repr | Age (in time steps) below which resources can’t reproduce | 4 (based on Mohr et al., (1973); Nechay et al., (1977)) |
| res_birth_type | Way in which new resources are added | Poisson sampling with lambda as the rate parameter |
| remove_pr | Density-independent resource mortality rate during a time step | Based on monthly survival rates (Table S2) |
| res_death_type | Density-independent removal | Resources are removed with a probability of remove_pr |
| max_ages | The highest age, in maximum number of time steps, a resource can reach | 24 (Nechay, 2000; Nechay et al., 1977; Weinhold & Kayser, 2006) |
| land_dim_1 | Landscape width (x-axis or number of cells horizontally) | 447 |
| land_dim_2 | Landscape height (y-axis or number of cells vertically) | 447 |
| res_move_type | Way in which the resource moves on the landscape | Uniform movement in any direction up to res_movement cells away from the original location, sampled from a uniform distribution |
| res_movement | Maximum number of cells the resource can move in any direction | 100 |

Table S2. Monthly survival rates for wild female European hamsters based on La Haye et al. (2020), as used in the natural resources submodel. These rates were calculated based on the weekly survival rates provided in the supplementary materials of the study.

| **Month** | **Survival rate** | **Month** | **Survival rate** |
| --- | --- | --- | --- |
| **January** | 0.976083968 | **July** | 0.767700722 |
| **February** | 0.948249690 | **August** | 0.842305069 |
| **March** | 0.902354400 | **September** | 0.877005333 |
| **April** | 0.811569975 | **October** | 0.891729601 |
| **May** | 0.805694059 | **November** | 0.939895976 |
| **June** | 0.794513672 | **December** | 0.956833861 |
| **YEAR** | 0.197332058 | | |

## Simulation scenarios

To explore a range of realistic model parameter choices, twelve different simulation scenarios were designed (see Table S5 for an overview).

**Scenario 1** was run with 100 repetitions, starting with an initial population size of 15 individuals. This simulation was the base scenario without any landscape changes, providing a reference point for subsequent comparisons. The initial population size of 15 reflects the estimated 30 hamsters remaining in the area, with approximately 50% being females, simulating a recent situation in Belgium (Smeets, 2022).

**Scenario 2** was identical to Scenario 1 but increased the number of repetitions to 1000 to assess the impact of repetition count on the model's outputs and the robustness of the base scenario results.

**Scenario 3** used the same parameters as Scenario 1, but with a larger initial population size of 250 individuals to evaluate the base scenario with a different starting population density, based on the goal of reaching a population of 500 individuals, which includes approximately 250 females.

**Scenario 4** was the same as Scenario 3 but with 1000 repetitions, paralleling the comparison approach used in Scenarios 1 and 2 to determine the effect of repetition count with a larger initial population size.

Each of the following scenarios started with an initial population of 250 individuals and ran for 100 repetitions. Scenarios 5 to 8 introduced variations in reproductive output or survival probability to assess their impacts on population dynamics.

**Scenario 5** introduced an increased lambda, averaging one extra litter per year. The formula for calculating lambda was adjusted by increasing the number of litters per year from 1.63 to 2.63, resulting in a lambda ≈ 3.83 female offspring per female per year. This was distributed over three months (June, July, and August) each with a lambda of 1.28. This simulation also included movement in August.

**Scenario 6** introduced an increased lambda, averaging three litters per year. The formula for calculating lambda was adjusted by increasing the number of litters per year from 1.63 to 3, resulting in a lambda ≈ 4.37 female offspring per female per year. This was distributed over three months (June, July, and August), each with a lambda of 1.46. This simulation also included movement in August.

**Scenario 7** introduced an increased lambda based on the mean annual reproduction rates before 1985 reported in Surov et al. (2016), with 2.56 litters per year and 8.24 pups per litter, resulting in a lambda ≈ 8.96 female offspring per female per year. This was distributed over three months (June, July, and August), each with a lambda of 3. This simulation also included movement in August.

**Scenario 8** introduced an increased mean annual survival of ca. 30%, with monthly survival rates as in La Haye et al. (2014) (Table S3). Other parameters remained the same as in the base scenario.

Table S3. Monthly survival rates for wild female European hamsters based on La Haye et al. (2014). These survival rates were used in Scenario 8 to simulate increased survival probability.

| **Month** | **Survival rate** | **Month** | **Survival rate** |
| --- | --- | --- | --- |
| **January** | 0.966 | **July** | 0.839 |
| **February** | 0.96 | **August** | 0.838 |
| **March** | 0.919 | **September** | 0.91 |
| **April** | 0.953 | **October** | 0.897 |
| **May** | 0.859 | **November** | 0.923 |
| **June** | 0.827 | **December** | 0.972 |
| **YEAR** | 0.2971 | | |

Scenarios 9 to 12 introduced variations in reproductive output and survival rates by incorporating custom functions. Different crop types are distributed among users while ensuring contiguous fields. This approach reflects real-world scenarios where users manage contiguous sections of the landscape with one type of crop. The landscape is distributed equally among all users. We specified beforehand how many users (i.e., fields) will be assigned a specific crop type, but distributed them randomly over the users.

The distribution of crop types is determined at the start of a simulation and remains the same throughout each time step. To model the ecological impacts of different crop types on the hamsters, a custom function adjusts population parameters for each individual based on the crop type present in the hamster’s cell within the GMSE landscape.

Three types of crops or agricultural practices were defined (Table S4). The landscape was divided among ten stakeholders, each receiving a contiguous part of the landscape. Four stakeholders were assigned crop type 1, another four received crop type 2, and the remaining two were given crop type 3. According to the Flemish species protection plan, some form of hamster-friendly management is required on at least 20%-25% of fields, leading to this distribution (Agentschap voor Natuur en Bos, 2015).

**Scenario 9** introduced an increased lambda of 3.83 in in crop type 3, similar to Scenario 5.

**Scenario 10** introduced an increased lambda of 4.37 in crop type 3, similar to Scenario 6.

**Scenario 11** introduced an increased lambda of 8.96 in crop type 3, similar to Scenario 7.

**Scenario 12** introduced an increased lambda of 3.83 combined with an increased yearly survival rate of ca. 30% in crop type 3, similar to Scenarios 5 and 8. This choice was made to investigate the effect of a simultaneous increase in reproduction, with one additional litter per year, alongside an increase in the survival rate. An increase in survival rate alone is neither expected nor considered sufficient.

Table S4. Parameter changes and rationale for individuals occupying cells with different crop types (as applied in Scenarios 9 to 12).

| **Crop type** | **Parameter change** | **Rationale** |
| --- | --- | --- |
| Crop type 1 – Unsuitable habitat | Individuals that occupy a crop type 1 cell have a survival probability of 0. | In reality, these include orchards, red cabbage, dairy farms, specialized pig farms, greenhouse vegetable cultivation and any other fields that lack sufficient cover or food availability for hamsters, making them unsuitable habitats (Bald et al., 2021; Müskens et al., 2019). |
| Crop type 2 – Conventional crops and agricultural practices | None – base scenario | These are crops where hamsters can survive and find cover and food, but early harvesting removes cover and food while the productive season is still ongoing. Suitable crops include cereals, alfalfa, and specialty crops like carrots (Bald et al., 2021; Kuiters et al., 2010; Müskens et al., 2019; Out et al., 2011). |
| Crop type 3 – Hamster-friendly agriculture | An increased lambda in June, July, and August of 1.28 in Scenario 9 and 12, 1.46 in Scenario 10, and 3 in Scenario 11. Increased survival rates as shown in Table S3 in Scenario 11. | According to Kuiters et al. (2010), hamster-friendly agriculture is characterised by (1) crops with at least 80% cover from April 15^th^ to September 15^th^, (2) being well-passable at ground level, (3) while also offering enough ground-level cover to protect against ground predators. This includes late or no harvesting to allow hamsters to wean an extra litter, the cultivation of crops that provide food and cover for the hamster (e.g., winter wheat, spring wheat, and alfalfa), crop rotation, and planting herb-rich grass strips. |

Table S5. Overview of parameter differences in all simulation scenarios.

| **Scenario** | **Repetitions** | **Initial population size** | **Lambda (♀/♀/year)** | **Survival rate (%/year)** | **Landscape changes** |
| --- | --- | --- | --- | --- | --- |
| 1 | 100 | 15 | 2.38 | 20 | No |
| 2 | 1000 | 15 | 2.38 | 20 | No |
| 3 | 100 | 250 | 2.38 | 20 | No |
| 4 | 1000 | 250 | 2.38 | 20 | No |
| 5 | 100 | 250 | 3.83 | 20 | No |
| 6 | 100 | 250 | 4.37 | 20 | No |
| 7 | 100 | 250 | 8.96 | 20 | No |
| 8 | 100 | 250 | 2.38 | 30 | No |
| 9 | 100 | 250 | Crop type 2: 2.38  Crop type 3: 3.83 | Crop type 1: 0  Crop type 2-3: 20 | Yes |
| 10 | 100 | 250 | Crop type 2: 2.38  Crop type 3: 4.37 | Crop type 1: 0  Crop type 2-3: 20 | Yes |
| 11 | 100 | 250 | Crop type 2: 2.38  Crop type 3: 8.96 | Crop type 1: 0  Crop type 2-3: 20 | Yes |
| 12 | 100 | 250 | Crop type 2: 2.38  Crop type 3: 3.83 | Crop type 1: 0  Crop type 2: 20  Crop type 3: 30 | Yes |

## References

Agentschap voor Natuur en Bos. (2015). *Soortbeschermingsprogramma voor de Europese hamster in Vlaanderen, 2015- 2020*. Agentschap voor Natuur en Bos.

Bach, A., Minderman, J., Bunnefeld, N., Mill, A. C., & Duthie, A. B. (2022). Intervene or wait? A model evaluating the timing of intervention in conservation conflicts adaptive management under uncertainty. *Ecology and Society*, *27*(3), art3. https://doi.org/10.5751/ES-13341-270303

Bald, V., Boetzl, F. A., & Krauss, J. (2021). Where do hamsters go after cereal harvest? A case study. *Basic and Applied Ecology*, *54*, 98-107. https://doi.org/10.1016/j.baae.2021.04.008

Bunnefeld, N., Hoshino, E., & Milner-Gulland, E. J. (2011). Management strategy evaluation: A powerful tool for conservation? *Trends in Ecology & Evolution*, *26*(9), 441-447. https://doi.org/10.1016/j.tree.2011.05.003

Cusack, J. J., Duthie, A. B., Minderman, J., Jones, I. L., Pozo, R. A., Rakotonarivo, O. S., Redpath, S., & Bunnefeld, N. (2020). Integrating conflict, lobbying, and compliance to predict the sustainability of natural resource use. *Ecology and Society*, *25*(2), art13. https://doi.org/10.5751/ES-11552-250213

Descamps, S., & Ramaekers, B. (2020). *Eindverslag monitoring gezenderde Europese hamsters (Cricetus cricetus) te Widooie: Mei—Oktober 2020*. PXL Bio-Research.

Duthie, A. B., Cusack, J. J., Jones, I. L., Minderman, J., Nilsen, E. B., Pozo, R. A., Rakotonarivo, O. S., Van Moorter, B., & Bunnefeld, N. (2018). GMSE: An r package for generalised management strategy evaluation. *Methods in Ecology and Evolution*, *9*(12), 2396-2401. https://doi.org/10.1111/2041-210X.13091

Górecki, A. (1977). Energy flow through the common hamster population. *Acta Theriologica*, *22*, 25-66. https://doi.org/10.4098/AT.arch.77-2

Grimm, V., Berger, U., Bastiansen, F., Eliassen, S., Ginot, V., Giske, J., Goss-Custard, J., Grand, T., Heinz, S. K., Huse, G., Huth, A., Jepsen, J. U., Jørgensen, C., Mooij, W. M., Müller, B., Pe’er, G., Piou, C., Railsback, S. F., Robbins, A. M., … DeAngelis, D. L. (2006). A standard protocol for describing individual-based and agent-based models. *Ecological Modelling*, *198*(1-2), 115-126. https://doi.org/10.1016/j.ecolmodel.2006.04.023

Grimm, V., Railsback, S. F., Vincenot, C. E., Berger, U., Gallagher, C., DeAngelis, D. L., Edmonds, B., Ge, J., Giske, J., Groeneveld, J., Johnston, A. S. A., Milles, A., Nabe-Nielsen, J., Polhill, J. G., Radchuk, V., Rohwäder, M.-S., Stillman, R. A., Thiele, J. C., & Ayllón, D. (2020). The ODD Protocol for Describing Agent-Based and Other Simulation Models: A Second Update to Improve Clarity, Replication, and Structural Realism. *Journal of Artificial Societies and Social Simulation*, *23*(2), 7. https://doi.org/10.18564/jasss.4259

Hemming, V., Camaclang, A. E., Adams, M. S., Burgman, M., Carbeck, K., Carwardine, J., Chadès, I., Chalifour, L., Converse, S. J., Davidson, L. N. K., Garrard, G. E., Finn, R., Fleri, J. R., Huard, J., Mayfield, H. J., Madden, E. M., Naujokaitis-Lewis, I., Possingham, H. P., Rumpff, L., … Martin, T. G. (2022). An introduction to decision science for conservation. *Conservation Biology*, *36*(1), e13868. https://doi.org/10.1111/cobi.13868

Kayser, A. (2001). Aspekte der Raum- und Baunutzung beim Feldhamster. *Jahrbuchern des Nassauischen Vereins für Naturkunde*, *122*, 149-151.

Kuiters, A. T., La Haye, M. J. J., Muskens, G. J. D. M., & Van Kats, R. J. M. (2010). *Perspectieven voor een duurzame bescherming van de hamster in Nederland*.

La Haye, M. J. J., Swinnen, K. R. R., Kuiters, A. T., Leirs, H., & Siepel, H. (2014). Modelling population dynamics of the Common hamster (Cricetus cricetus): Timing of harvest as a critical aspect in the conservation of a highly endangered rodent. *Biological Conservation*, *180*, 53-61. https://doi.org/10.1016/j.biocon.2014.09.035

La Haye, M. J. J., van Kats, R. J. M., Müskens, G. J. D. M., Hallmann, C. A., & Jongejans, E. (2020). Predation and survival in reintroduced populations of the Common hamster Cricetus cricetus in the Netherlands. *Mammalian Biology*, *100*(6), 569-579. https://doi.org/10.1007/s42991-020-00063-5

Millesi, E., Winkler, H., & Hengsberger, R. (2008). *The Common Hamster (Cricetus cricetus): Perspectives on an endangered species* (p. 0xc1aa5576_0x001c97b9). Verlag der Österreichischen Akademie der Wissenschaften. https://doi.org/10.1553/0x001c97b9

Mohr, U., Schuller, H., Reznik, G., Althoff, J., & Page, N. (1973). *Breeding of European hamsters.* https://www.cabidigitallibrary.org/doi/full/10.5555/19740108882

Müskens, G. J. D. M., La Haye, M. J. J., Van Kats, R. J. M., Schrijver, R., Elbersen, H. W., Heldens, R., & Kuiters, A. T. (2019). *Hamster op eigen benen: Ervaringen met alternatieve beheerpakketten 2015-2018*. Wageningen Environmental Research. https://doi.org/10.18174/502204

Nechay, G. (2000). *Status of Hamsters Cricetus Cricetus, Cricetus Migratorius, Mesocricetus Newtoni, and Other Hamster Species in Europe*. Council of Europe.

Nechay, G., Hamar, M., & Grulich, I. (1977). The Common Hamster (Cricetus cricetus [L.]); a Review. *EPPO Bulletin*, *7*(2), 255-276. https://doi.org/10.1111/j.1365-2338.1977.tb02727.x

Nilsson, L., Bunnefeld, N., Minderman, J., & Duthie, A. B. (2021). Effects of stakeholder empowerment on crane population and agricultural production. *Ecological Modelling*, *440*, 109396. https://doi.org/10.1016/j.ecolmodel.2020.109396

Out, M., Van Kats, R. J. M., Kuiters, L., Müskens, G. J. D. M., & La Haye, M. J. J. (2011). Hard to stay under cover: Seven years of crop management aiming to preserve the Common hamster (Cricetus cricetus) in the Nether- lands. *Säugetierkundliche Informationen*, *Jena 8*, 37-49.

Smeets, J. (2022, augustus 1). Ondanks veel overheidsgeld blijft populatie wilde hamsters klein: ‘Transitie van landbouw is nodig’. *VRT NWS*. https://www.vrt.be/vrtnws/nl/2022/08/01/wilde-hamsters/

Surov, A., Banaszek, A., Bogomolov, P., Feoktistova, N., & Monecke, S. (2016). Dramatic global decrease in the range and reproduction rate of the European hamster Cricetus cricetus. *Endangered Species Research*, *31*, 119-145. https://doi.org/10.3354/esr00749

Van Donink, S., & Baert, K. (2023). *De Europese hamster (Cricetus cricetus) in Nederland—Evaluatie van 25 jaar hamsterbescherming en -beleid*. Instituut voor Natuur- en Bosonderzoek. https://doi.org/10.21436/inbor.93612311

Van Wijk, R., La Haye, M. J. J., Van Kats, R. J. M., & Muskens, G. J. D. M. (2011). Movement characteristics of the Common hamster (Cricetus cricetus) in Limburg, the Netherlands. In *Proceedings of the 16t and 17th Meeting of the International Hamster Workgroup; Ranis, Germany (2009), Gödollo, Hungary (2010)* (pp. 79-92). https://research.wur.nl/en/publications/movement-characteristics-of-the-common-hamster-cricetus-cricetus-

Weinhold, U., & Kayser, A. (2006). *Der Feldhamster: Cricetus cricetus* (1. Aufl). Westarp-Wissenschaften.
